# Research progress on the interaction between glucose metabolic reprogramming and lactylation in tumors

**DOI:** 10.3389/fimmu.2025.1595162

**Published:** 2025-07-14

**Authors:** Yi Yang, Yi Wu, Hui Chen, Zehai Xu, Ruisi Lu, Sijie Zhang, Rui Zhan, Qinghua Xi, Yunfeng Jin

**Affiliations:** ^1^ Department of Obstetrics and Gynecology, Affiliated Hospital of Nantong University, Nantong, China; ^2^ Department of Clinical Medicine, Medical School of Nantong University, Nantong, China; ^3^ Department of Laboratory Medicine, Affiliated Hospital of Nantong University, Nantong, China

**Keywords:** glucose metabolic reprogramming, lactylation, lactate, tumor microenvironment, immune cells, macrophage, posttranslational modification

## Abstract

Glucose metabolic reprogramming describes the alterations in intracellular metabolic pathways in response to variations in the body’s internal environment. This metabolic reprogramming has been the subject of extensive research. The primary function is to enhance glycolysis for rapid ATP production, even with sufficient oxygen, leading to a significant accumulation of lactic acid, which subsequently affects the functions of tumor cells and immune cells within TME. Lactylation represents a newly identified post-translational modification (PTM) that occurs due to lactate accumulation and is observed in various proteins, encompassing both histone and non-histone types. Lactylation alters the spatial configuration of proteins, influences gene transcription, and thereby regulates gene expression. This modification serves as a significant epigenetic regulatory factor in numerous diseases. Glucose metabolic reprogramming and lactylation are intricately linked in the process of tumorigenesis. Glucose reprogramming activates essential enzymes, including hexokinase 2 (HK2), pyruvate kinase M2 (PKM2), and lactate dehydrogenase A (LDHA), through transcription factors such as HIF-1α and c-Myc, thereby enhancing glycolysis and lactate accumulation. Lactate functions as a metabolite and signaling molecule, acting as a substrate for lactylation facilitated by histone acetyltransferases such as CBP/p300. This epigenetic modification inhibits antitumor immunity through the upregulation of oncogenic signaling pathways, the induction of M2-type macrophage polarization, and the dysfunction of T-cells. Glucose metabolic reprogramming not only influences lactate synthesis but also provides sufficient substrates for lactate modification. The two factors jointly affect gene expression and protein function, acidify the tumor microenvironment, regulate immune evasion, and promote carcinogenesis. This review systematically details the mechanisms of lactylation and glucose metabolic reprogramming, their impacts on immune cells within the tumor microenvironment, and their interrelations in tumor progression, immunity, and inflammation.

## 1 Introduction

**Figure 1:**
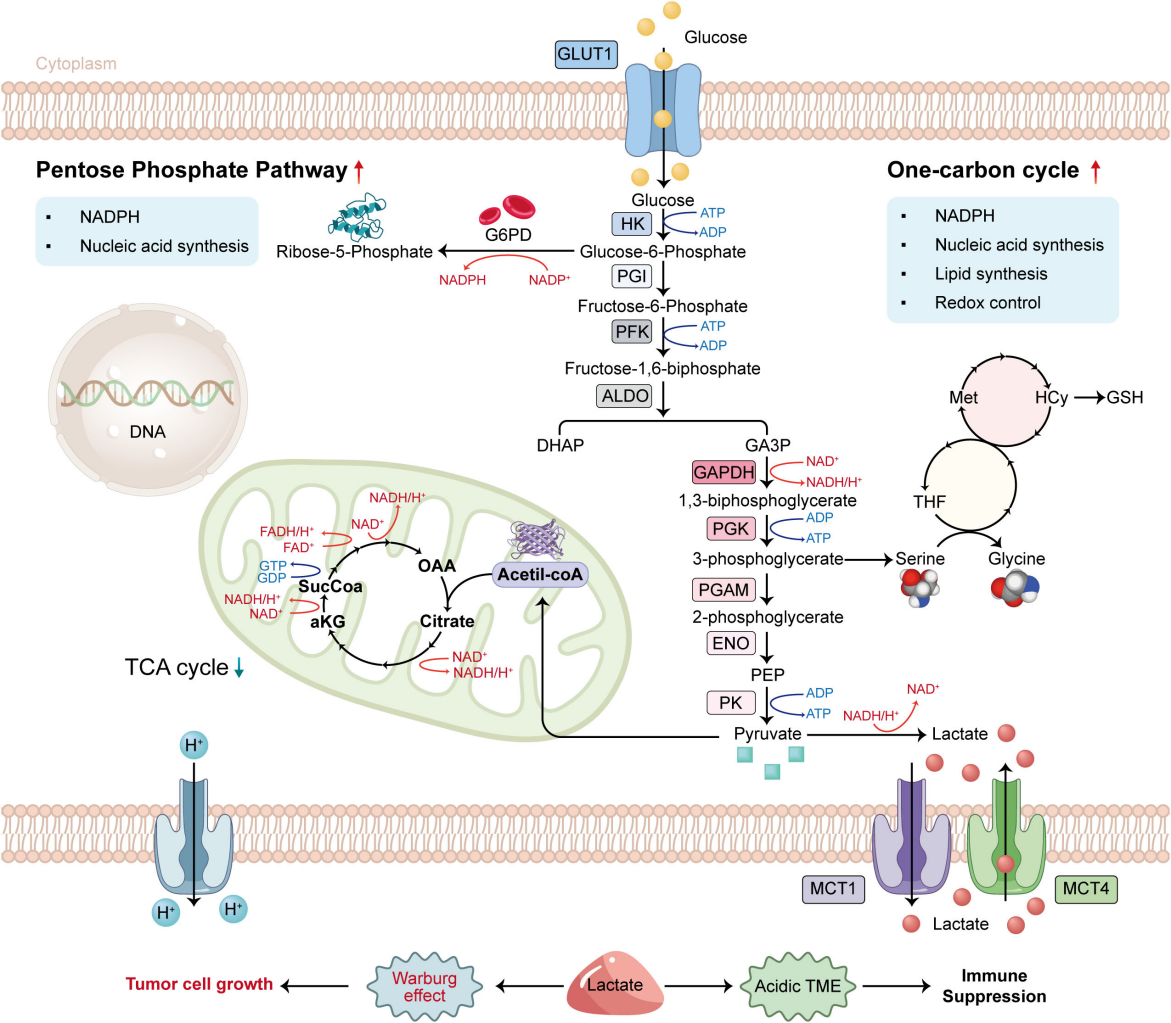
Diagram of glucose metabolism in tumor cells.

Glucose metabolic reprogramming is a characteristic feature of tumor cells. Cells exhibit a preference for glycolysis, an inefficient ATP production pathway, even when sufficient oxygen is available for aerobic respiration. The Warburg effect describes this phenomenon (1). The glycolysis pathway involves the uptake of glucose facilitated by glucose transporter 1 (GLUT1) and the conversion of glucose into pyruvate through a series of 10 sequential enzymatic reactions that are anaerobic in nature. A fraction of the newly produced pyruvate is converted into Acetyl-CoA to facilitate the TCA cycle, while the rest is reduced to lactic acid by lactate dehydrogenase (LDH) to regenerate NAD+, thereby enabling glycolysis to persist and preserving the redox equilibrium of NAD+/NADH (2). Monocarboxylate transporters (MCTs) facilitate the cellular export of surplus lactate. Glycolysis not only generates energy but also yields biosynthetic precursors essential for various metabolic pathways. 3-Phosphoglyceric acid is utilized in the one-carbon pathway, which is significant in nucleotide and amino acid synthesis (3). Glucose metabolic reprogramming plays a critical role in the metabolism of tumor cells, facilitating tumor formation and progression (**Figure 1**). Lactic acid produced through glucose metabolic reprogramming contributes to an acidic tumor microenvironment, facilitating tumor cell invasion and metastasis while inhibiting immune cell functions. It can affect T cell function and assist tumor cells in evading the immune response (4).

Lactylation represents a distinct post-translational modification of proteins closely associated with glycolytic reprogramming. Lactate-induced lysine lactylation (Kla) may serve as a significant connection between epigenetics and metabolic reprogramming (5). Lysine lactylation is a PTM generated by lactate buildup that involves the covalent modification of lysine residues in proteins with lactyl groups using specific enzymes, altering protein functions, activities, and interactions with other biomolecules (6). Lactate, previously considered a metabolic byproduct, is now understood to be transported into cells through the monocarboxylate transporter 1 (MCT1), where it is converted to lactyl-CoA. This conversion influences associated proteins and regulates the expression of chromatin genes. Lactate serves a significant function in cellular regulation as a signaling molecule (7). Recent research indicates that lactylation plays a vital role in various functions, such as regulating immune escape, modulating immune cell metabolism, and promoting angiogenesis.

Recent research indicates that lactylation modification is governed by writer enzymes, including CBP/p300, and eraser enzymes, such as HDAC1-3. It is essential in facilitating angiogenesis, modulating immune cell metabolism, and enabling immune evasion, among other functions. Lactylation regulates glycolytic enzymes such as LDHA and HK2 via histone modification, creating a feedforward loop associated with metabolic reprogramming. H3K18 lactylation in pancreatic cancer cells promotes the expression of TTK/BUB1B kinase, which subsequently upregulates LDHA, leading to increased lactate production. Nonetheless, significant gaps persist: (1) the context-specific functions of CBP/p300 and HDAC1–3 across tumor subtypes; (2) the determination of lactylation as a causal epigenetic factor versus a passive response to metabolic stress; and (3) the interplay between lactylation and acetylation in the functionality of immune cells. This review systematically examines the mechanisms and relationships between lactylation and glycolytic reprogramming. To elucidate the complex relationship between lactylation and glycolytic reprogramming, we integrate lactylation with the conventional metabolic reprogramming observed in malignant tumors. This will provide a foundation for future research into their roles in malignant tumor development and inspire novel therapeutic strategies.

The traditional pathway of the Warburg effect involves the conversion of glucose to lactate. Pyruvate generated from aerobic glycolysis subsequently enters the tricarboxylic acid cycle, thereby inhibiting the Warburg effect. The enhancement of the glycolytic pathway results in elevated lactate and proton secretion in the extracellular space, as well as an increased availability of glycolytic intermediates. This fact facilitates the flow to the pentose phosphate pathway (PPP) and the single carbon cycle, thereby promoting nucleotide and lipid synthesis essential for maintaining redox homeostasis.

## 2 Glucose metabolic reprogramming

Glucose metabolic reprogramming refers to the adaptive alterations of the body’s glycolytic pathways in response to changes in the internal environment. Cells generally utilize the tricarboxylic acid cycle pathway for glucose metabolism. Several factors, including the hypoxic conditions of tumor cells, the inflammatory state of the body, and the activation of oncogenic signaling pathways (e.g., MYC and PI3K) (8), influence the quantity of glucose transporters and the expression and activity levels of key glycolysis-related enzymes (such as phosphofructokinase and hexokinase), thereby affecting the glycolytic rate. Consequently, functions such as energy production and metabolic product synthesis are adjusted to meet the requirements of cells within a specific environment (9). In the 1920s, German biologist Otto Warburg first proposed the phenomena observed in tumor cells, which is referred to as the Warburg effect (1). Cancer cells preferentially convert glucose to lactate for ATP production, even in the presence of sufficient oxygen, rather than efficiently utilizing the OXPHOS pathway for energy generation. Upon activation, T cells undergo metabolic reprogramming, transitioning from mitochondrial oxidative phosphorylation to glycolysis-dependent metabolism to meet the organism’s requirements. Cells utilize glucose metabolic reprogramming to maintain survival and essential functions during stress conditions, including hypoxia, ischemia, or specific metabolic disorders (10).

### 2.1 The role of glucose metabolic reprogramming

Glucose metabolic reprogramming enables tumor cells to rapidly adjust to a nutrient-poor environment while simultaneously modulating immune cells, thereby inhibiting tumor immunological activity. It plays a significant role in cancer progression (11). Recent studies indicate that levels of local oxygen, glucose, and other nutrients in malignant tumors are significantly lower than those in corresponding normal tissue cells. Tumor cells alter their glucose metabolism to adapt to hypoxic and nutrient-deficient conditions, including a shortage of glucose (12). Lactate generated through glycolytic reprogramming establishes an acidic extracellular environment, which inhibits the proliferation of immune cells, induces the death of tumor-infiltrating immune cells, and facilitates the polarization of macrophages towards the M2 phenotype. These mechanisms significantly contribute to tumor genesis, metastasis, and immune evasion (13). Glucose metabolic reprogramming plays a crucial role in the host’s immunological response and inflammation (9).

Glucose metabolic reprogramming induces macrophages to upregulate essential glycolytic molecules, including glucose transporter type 1 (GLUT1), phosphofructokinase (PFK), pyruvate dehydrogenase kinase isozyme 1 (PDK1), and lactate dehydrogenase A (LDHA) (5). The upregulation promotes the transition of macrophages to the M1 phenotype, enhancing the expression of pro-inflammatory cytokines and contributing to inflammation progression and development. Inhibiting glucose metabolic reprogramming generally results in the transition of M1 macrophages to the M2 phenotype, which encourages tumor-associated macrophages to induce immune suppression, closely linked to tumor immune evasion processes. Glucose metabolic reprogramming influences the antigen processing and presentation capabilities of macrophages and dendritic cells (DCs) to T cells, thereby participating in host defense mechanisms. The degree of antigen presentation by dendritic cells correlates directly with glycolysis activity. The expression of Major Histocompatibility Complex (MHC) class I and class II, crucial for dendritic cell activation of CD4+ T cells and CD8+ T cells, is directly affected by glycolysis levels (14).

#### 2.1.1 Glucose metabolic reprogramming in inflammation and immunity

Glucose metabolic reprogramming has a vital function in tumor immunity and inflammation. Macrophages are essential components of the innate immune system and play a crucial role in the inflammatory response. Glucose metabolic reprogramming can alter the phenotype of macrophages, hence modulating inflammation (15).

Under inflammatory conditions, M1 macrophages undergo significant glucose metabolic reprogramming to sustain their proinflammatory activities. This process involves the upregulation of glucose transporter 1 (GLUT1) and key glycolytic enzymes including hexokinase 2 (HK2), phosphofructokinase (PFK), and lactate dehydrogenase A (LDHA), which are primarily mediated by NF-κB and STAT3 signaling pathways. A representative study by Xu et al. (16) demonstrated that IL-6 modulates glucose metabolism through formation of a molecular complex comprising STAT3, HK2, and voltage-dependent anion channel 1 (VDAC1), ultimately promoting glycolytic and lactate generation. This metabolic shift further sustains the production of proinflammatory cytokines including TNF-α and IL-6, which is a process essential for both antimicrobial defense and tumor immune surveillance. As inflammation progresses, certain immune cells undergo dynamic metabolic adaptations, with some macrophages transitioning from the M1 to M2 phenotype (17), shifting their metabolic profile toward pathways that support tissue repair processes.

M2 macrophages have reduced glycolysis and preferential use of oxidative phosphorylation. The metabolic profile is characterized by reduced levels of GLUT1 and LDHA, alongside heightened activity of enzymes associated with the tricarboxylic acid (TCA) cycle and mitochondrial respiration. Lactate generated by adjacent tumor cells or fibroblasts is absorbed by M2 macrophages through MCT1, which activates NF-κB signaling and enhances the expression of anti-inflammatory genes such as Arg1 and IL-10. This metabolic reprogramming facilitates tissue repair and enables tumor immune evasion. M2 macrophages suppress T cell activation and promote angiogenesis through PPAR-γ signaling pathways. The differing glycolytic activity of M1 and M2 macrophages highlights their distinct functions in tumor immunity. M1 cells facilitate proinflammatory tumor rejection, while M2 cells create an immunosuppressive environment that supports tumor growth. Blocking glycolytic reprogramming typically leads to the transformation of M1 macrophages into M2 macrophages, thereby facilitating the development of tumor-associated macrophages that exhibit immunosuppression and contribute to tumor immune evasion. In the initial phase of inflammation, the activation of the immune system leads to the activation of T cells, which undergo glycolytic reprogramming. This process involves a transition from mitochondrial oxidative phosphorylation to glycolysis for energy production. Such a shift enables T cells to efficiently support rapid proliferation, differentiation, and the secretion of cytokines, thereby enhancing their role in the immune response and pathogen elimination (18). The primary metabolism of T cells is characterized by low-rate catabolism, generating ATP via pyruvate and fatty acid oxidation. Upon activation, T cell metabolism undergoes reprogramming to support cell growth and clonal expansion, primarily generating ATP through glycolysis and supplying raw materials for biosynthesis. Previous studies indicate that T cell-specific knockout of the Slc2a1 gene, which encodes GLUT1, diminishes glucose uptake and glycolysis, subsequently impairing the proliferation and differentiation of CD4 + T cells (16).

#### 2.1.2 The impact of glucose metabolic reprogramming on the tumor microenvironment

TME refers to the internal conditions that facilitate the development, proliferation, and metastasis of tumor cells. The composition primarily includes tumor cells, adjacent immune cells, cancer-associated fibroblasts (CAFs), a limited extracellular matrix, and cytokines (17). CAFs are located in proximity to tumors, exhibit unique genetic characteristics, and can affect tumor initiation, invasion, and progression through the secretion of cytokines, chemokines, and angiogenic factors. CAFs, similar to tumor cells, experience metabolic reprogramming, shifting to a more glycolytic phenotype and producing metabolic byproducts such as lactate, pyruvate, and ketone bodies, which supply energy to adjacent tumor cells. Metabolic reprogramming is associated with the acidification of the tumor microenvironment, a fundamental characteristic of tumor growth (19).

TME is defined by a multifaceted and dynamic metamorphosis that influences both the biological functions of tumor cells and their responses, and is closely linked to the onset, progression, metastasis, and therapeutic responses of malignancies. Cancer cells exhibiting abnormal metabolism use excessive oxygen and nutrients, leading to hypoxia and an accumulation of metabolic byproducts in the tumor microenvironment. Lactate produced from tumors can polarize tumor-associated macrophages (TAMs) into an anti-inflammatory and immunosuppressive state, hence enhancing tumor survival and growth. Conversely, macrophage polarization can induce metabolic changes inside macrophages (20). Increased metabolites in the tumor microenvironment, especially lactate, provide an immunosuppressive environment that enhances cancer cell proliferation and facilitates immune evasion (21). Lactate serves as an essential signaling molecule as well as an energy substrate. Lactate dehydrogenase (LDH) levels serve as a diagnostic for unfavorable prognosis and the effectiveness of immunotherapy, while serum lactate concentration correlates positively with tumor burden (22). Lactate, a mediator, stimulates the production of pro-inflammatory cytokines, including IL-23 and IL-17, in immune cells that infiltrate tumors, hence enhancing carcinogenesis and diminishing anti-tumor activity. Lactate is essential in establishing an immunosuppressive milieu that facilitates tumor cell proliferation and inhibits the functionality of immune cells as signaling molecules inside TME. It also inhibits immune cell activation by obstructing their glycolytic reprogramming (23).

### 2.2 Molecular mechanisms of glucose metabolic reprogramming

Glucose metabolic reprogramming is controlled by several variables. Key enzyme regulation, transcription factor activity, signaling pathway effect, and metabolite feedback regulation are some of its primary mechanisms. The key enzymes in glucose metabolic reprogramming mainly include hexokinase (HK2), pyruvate kinase M2(PKM2), lactate dehydrogenase A(LDHA) and so on. Previous studies have shown that hypoxic response elements (HRE) in the promoter region of the HK2 gene can bind directly to HIF-1 and activate HK2 transcription, thereby promoting the first step of glycolysis (24). The high expression of HK2 allows tumor cells to maintain high glycolytic activity even under normoxic conditions, supporting the energy and biosynthetic precursors required for rapid proliferation. Studies have found that in drug-resistant ovarian cancer (25), PKM2 can bind to the c-Myc promoter region to determine the metabolic direction of pyruvate, thereby up-regulating glycolysis and promoting the proliferation and drug resistance of cancer cells. LDHA can catalyze the generation of pyruvate into lactic acid and maintain the continuous glycolysis. Transcription factors (HIF-1, c-Myc, p53, NF-κB, etc.) also regulate glucose metabolic reprogramming and promote tumor development. HIF-1α is the core regulator of glucose metabolic reprogramming under hypoxia conditions. It directly binds to the hypoxia response element (HRE) of glycolytic related genes (GLUT1, HK2, PDK1, LDHA, etc.) to promote its expression and inhibit mitochondrial oxidative metabolism. In order to coordinate the actions of several transcription factors and pathway components, HIF-1 serves as a signaling hub. According to research by MA (26) and others, CREB1 drives the malignant behavior of ovarian tumor cells by upregulating WNK1, which in turn drives the development of ovarian cancer by upregulating HIF-1 expression. The proto-oncogene c-Myc binds to the E-box sequence of the promoter of LDHA, HK2, PKM2 and other genes to directly promote its transcription. NF-κB promotes glycolysis and lactic acid production through inflammatory signaling, supporting tumor cell survival. Similarly, the occurrence of glycolytic reprogramming is cooperatively regulated by several signaling pathways. ACTL6A is overexpressed in ovarian cancer, according to Zhang (27) and colleagues. This activates the ACTL6A/PGK1 signaling pathway, which is controlled by follicle-stimulating hormone (FSH) via the PI3K/AKT pathway. Phosphoglycerate kinase 1(PGK1) expression can be upregulated by overexpressing ACTL6A, but PGK1 expression is downregulated by ACTL6A knockdown, which impacts the glycolysis and growth of ovarian cancer cells. To summarize the primary molecular pathways of glycolytic reprogramming, we present the findings of different researchers’ studies.

## 3 Lactylation

**Figure 2:**
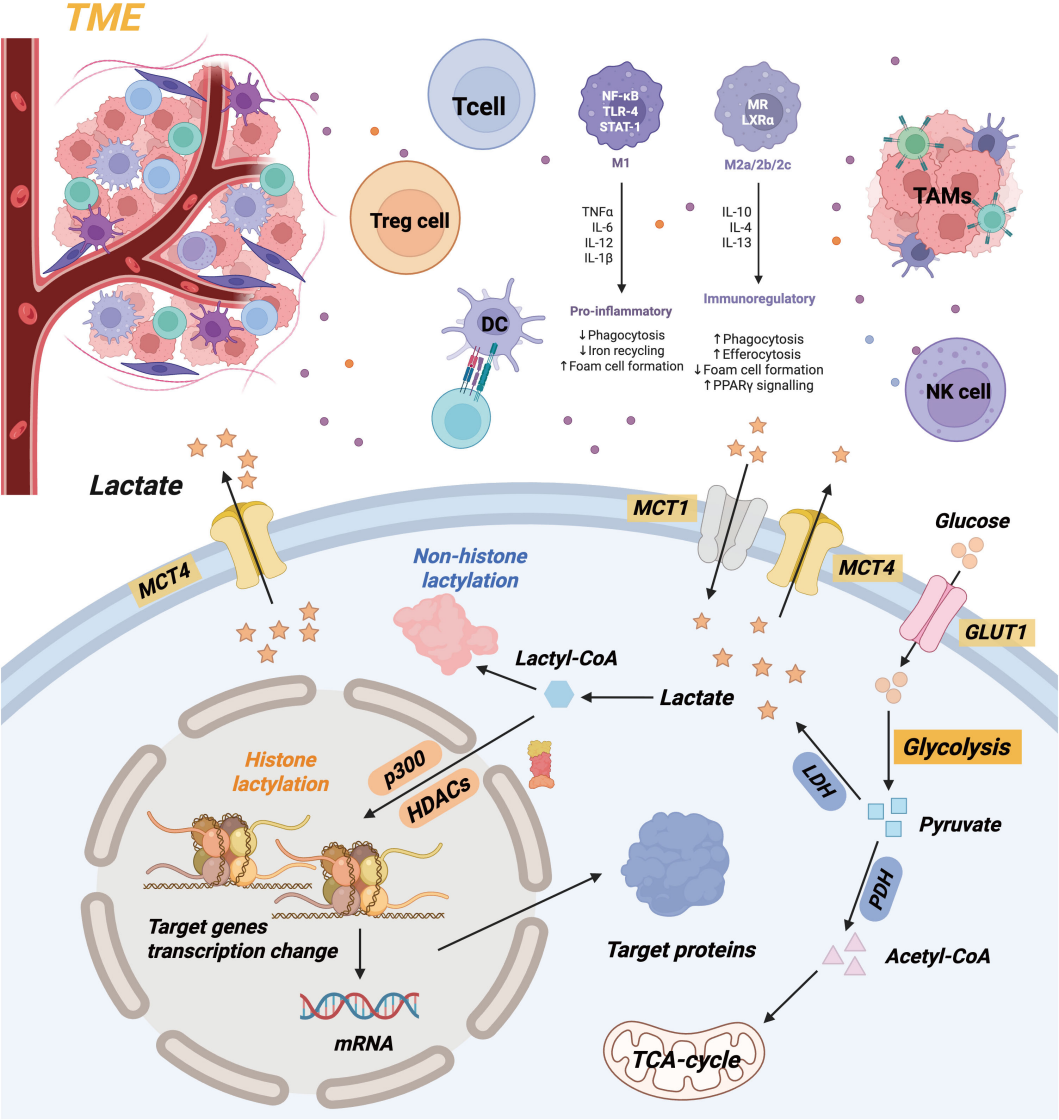
Mechanism diagram of lactate metabolism and lactate’ effects in TME.

Lactate, a consequence of glycolysis, is essential for immune regulation, signaling molecules, and energy supply (7). Lactate was erroneously categorized as a metabolic byproduct for an extended duration. Under hypoxic conditions, a series of enzyme reactions converts glucose into pyruvate upon its entry into the cytoplasm. The glycolysis pathway is initiated by hypoxia’s suppression of the tricarboxylic acid cycle, with lactate dehydrogenase (LDH) directly converting pyruvate to lactate (28). The Zhao Yingming team initially proposed lactylation modification in 2019. It delineates a post-translational modification method utilizing lysine and lactate residues as substrates for protein modification. The team’s findings establish a foundation for understanding metabolic reprogramming in cancer initiation and development by showing that cancer cells preferentially convert glucose to lactate, even in aerobic conditions (29). Lactylation is a notable post-translational modification that can influence the stability, interactions, and functions of proteins. Both histones and non-histones in the body can undergo modifications, enabling their involvement in processes such as regulating gene transcription and initiating macrophage polarization. Consequently, lactylation research has recently attracted significant attention (**Figure 2**).

Lactate may be generated intracellularly by glycolysis or absorbed from extracellular plasma. Intracellular lactate can be transformed into lactoyl-CoA and participates in the lactylation of histone and non-histone proteins. The buildup of lactate in TME can alter the phenotypic of immune cells. Lactate impedes the proliferation and cytotoxic function of T cells and NK cells, while diminishing the development of dendritic cells. Lactate furthermore facilitates the polarization of tumor-associated macrophages (TAMs) towards pro-tumor phenotypes, consequently enhancing tumor development and invasion.

### 3.1 The role of lactylation modification

Lactylation alteration influences various physiological and pathological processes. This encompasses immune cell functions (including macrophage polarization and the secretion of inflammatory mediators), cellular metabolism (influencing energy and material metabolism through the modulation of critical enzymes), tumorigenesis (such as metabolic reprogramming of tumors, regulation of the immune microenvironment, tumor angiogenesis, and metastasis), and cell signal transduction (regulating protein activities and interactions while participating in intracellular signaling) (30). Lactylation alteration is pivotal in the initiation and advancement of specific diseases.

#### 3.1.1 Lactylation modification participates in the regulation of immunity and inflammation

Lactylation modification is a significant element in macrophage polarization. Research indicates that (31) lactate accumulates in macrophages when they polarize into the M1 phenotype, with a significant increase in histone lactylation during the later phases of this polarization. Histone Kla can modulate the expression of cell-repair genes, such as Arg1, to polarize pro-inflammatory M1 macrophages into anti-inflammatory M2 macrophages, thereby affecting the trajectory and outcome of the inflammatory response and promoting the resolution of inflammation after inflammatory or anti-inflammatory activation (9).

Moreover, alterations in lactylation can affect T cell metabolic reprogramming, rendering them more inclined towards glycolytic metabolism and so impacting immunological functions. Regulatory T cells (Tregs), a subset of T lymphocytes characterized by the expression of the transcription factor FOXP3, are essential for immunological tolerance, homeostasis, and tumor immune evasion (32). The quantity of lactate-modified Treg cells within TME increases, along with their functionalities. They can inhibit the function of tumor-specific effector T cells, enabling tumor cells to evade the immune system. A recent study (33) indicates that the tumor metabolite lactate can modulate the lactylation of the MOESIN protein at the Lys72 residue in Treg cells. This enhances the connection between MOESIN and the TGF-I receptor I, as well as downstream Smad3 signaling, hence augmenting the functions of Treg cells and facilitating immunosuppression in the tumor microenvironment.

Besides the aforementioned pathophysiological implications, lactylation’s regulatory function in cancer has garnered significant attention. Moreover, lactic acid will influence the functionality of NK cells, thereby hindering the release of IFN-as Research on mechanisms indicates that excessive consumption of pathological levels of lactic acid by NK cells can lead to intracellular acidification and impede the up-regulation of the nuclear factor of activated T cells (NFAT) signaling pathway, thereby diminishing the production of NFAT-regulated IFN– and facilitating apoptosis (34). Significantly, both human and rat melanoma exhibit elevated levels of lactate. In immune-competent mice, diminishing lactic acid generation can impede tumorigenesis, while the infiltration of CD8+ T cells and NK cells secreting IFN-e within the tumor is markedly enhanced. In mice devoid of lymphocytes and NK cells, the tumorigenic potential exhibited minimal variation between the low lactate group and the control group (35). Recent study has revealed that lactate generated by the glycolysis of tumor cells within TME stimulates the mTOR pathway, resulting in the phosphorylation of the transcription factor TFEB and impeding its nuclear translocation. This alteration transforms tumor-associated macrophages (TAMs) within TME into immune cells that facilitate tumor proliferation. A negative connection between intratumoral lactate levels and overall survival in cervical cancer patients has been documented. Collectively, these data suggest that elevated lactate levels in the tumor and the resultant acidified TME will inhibit immune cell activity and undermine immunological monitoring of cancer, ultimately facilitating immune evasion (36). The elevated lactate levels in the internal environment can diminish the antigen-presenting capacity of dendritic cells (DC) and impair the anti-tumor immune response.

#### 3.1.2 The role of lactylation in tumor development

Tumors generate substantial lactate through the Warburg effect and glutamine hydrolysis, thereby activating the monocarboxylate transporters (MCTs) on the cell membrane. Tumor cells can rapidly extrude intracellular lactate to avert excessive intracellular acidification (37). This transport mechanism generates a “inverse pH gradient” between the intracellular and extracellular environments, resulting in an increase in internal pH and a decrease in external pH. This significantly elevates the acidity of TME, impacting immune cell function (38). TAMs, MSCDs, and Tregs in TME are all immunosuppressive. They facilitate tumor proliferation by suppressing anti-tumor immune responses and evading immune surveillance (39) (40). Preserving intracellular redox balance with an appropriate concentration of lactate protects tumor cells from oxidative stress, hence promoting their survival and proliferation. Lactylation can regulate the extracellular matrix. Cancer cells release metabolic byproducts, including lactate, which activate certain signaling pathways such as the PI3K/Akt pathway (41), to regulate cytoskeletal reorganization, the synthesis of cell adhesion molecules, and the function of matrix metalloproteinases. This facilitates the infiltration of cancer cells through the basement membrane, allowing them to infect adjacent tissues and metastasize to distant sites (42).

Lactate strongly influences the proliferation, metabolism, invasion, metastasis, and angiogenesis of tumor cells. Generally, tumor cells generate substantial quantities of lactic acid owing to their elevated glycolytic rate, which functions as an essential energy source for their survival and proliferation. Tumors utilize lactate in two distinct manners to generate energy (43). Lactate is utilized by cancer cells to interact with fibroblasts (CAFs). CAFs utilize lactate produced by aerobic glycolysis to facilitate the proliferation and dissemination of adjacent cancer cells. Secondly, the Warburg effect induces hypoxic cellular populations inside TME to generate substantial quantities of lactate. MCTs transport lactate to adjacent cells for oxidative phosphorylation (44). Lactate additionally has endocrine-like, paracrine, and autocrine activities inside the tumor microenvironment. Besides supplying sufficient energy for cancer via energy redistribution and effective use through the lactate shuttle mechanism, it also facilitates cancer invasion and metastasis by enhancing cell-to-cell interactions through the autocrine-paracrine signaling loop (45). Lactate can accelerate the development and metabolism of cancer cells by regulating key enzymes (46), including PDK-1 and LDH. LDHA and LDHB are the two subunits of the lactate dehydrogenase (LDH) enzyme group, which facilitate the reversible transformation of pyruvate into lactate. Essentially, LDHB oxidizes lactate to pyruvate, while LDHA preferentially reduces pyruvate to lactate and regenerates NAD+. Dysregulation of LDH levels, characterized by the overexpression of LDHA and the downregulation of LDHB, often promotes tumor proliferation (47). LDHA is a prospective target for oncological treatment, especially for malignancies reliant on the Warburg effect. Lactate activates HIF and c-Myc, leading to the expression of these two enzymes in cancer cells, enhancing aerobic glycolysis and ensuring sufficient energy for growth and proliferation in hypoxic conditions. Lactate promotes tumor development and invasion by inhibiting the degradation of HIF-1d and stimulating the synthesis of vascular endothelial growth factor (VEGF).

#### 3.1.3 Immune escape induced by lactate

Lactate and lactylation collaborate to circumvent anti-tumor immunity. Extracellular lactate decreases pH, hindering CD8+ T cell migration and NK cell cytotoxicity, whereas intracellular lactyl-CoA, produced from lactate, facilitates the lactylation of essential proteins. Lactate-induced lactylation of MOESIN at Lys72 in Tregs fortifies its association with TGF-c receptor I, hence augmenting Smad3 signaling and Treg suppressive efficacy. Tumor cells export lactate through MCT4, which is absorbed by macrophages, leading to H3K18 lactylation and M2 polarization, so establishing a pro-tumor feedback loop (96, 97).

### 3.2 Molecular mechanisms of lactylation

The generation and distribution of lactate, the regulation of “writer enzymes” and “eraser enzymes,” and the activation of signaling pathways constitute the three fundamental processes that dictate the extent of lactylation. Lactylation is regulated by various physiological factors that influence these essential processes.

#### 3.2.1 The impact of lactate production and transport on lactylation

Glycolysis is the principal pathway that generates lactate. Glucose is converted into pyruvate via a series of metabolic reactions under anaerobic or hypoxic conditions. LDH facilitates the conversion of pyruvate into lactate (48). A novel post-translational modification in the body is histone lactylation. Histone lactylation can be promoted by the external administration of sodium lactate and by increased endogenous lactate concentrations. Consequently, two significant factors affecting lactylation are lactate production and transport. The balance between glycolysis and mitochondrial metabolism is the principal factor influencing lactate production. The synthesis of lactate and, subsequently, the extent of lactylation in this process can be significantly influenced by alterations in the activity of key enzymes in the glycolytic pathway. Glycolysis accelerates, resulting in increased lactate production and enhanced lactylation, particularly when the activity of hexokinase, phosphofructokinase-1, and pyruvate kinase rise in response to heightened cellular energy demands. Lactate transport is another significant component affecting lactylation. MCTs are crucial in the lactate transport route (49). MCT4 predominantly transports intracellular lactate out of the cell, while MCT1 principally facilitates the influx of extracellular lactate into the cell. The dynamic equilibrium of lactate within and outside the cell is ensured by their existence. Under standard physiological conditions, MCT1 transports lactate into the cell as extracellular lactate concentrations increase, hence augmenting the availability of lactate for lactylation within the cell. Elevated MCT4 expression in tumor cells facilitates the retention of intracellular lactate, promotes the efflux of substantial quantities of endogenously produced lactate, and indirectly affects lactylation modification (50). The correct production and localization of MCT1 and MCT4 on the cell membrane can be facilitated by CD147, a co-chaperone protein of MCTs (51). The maintenance of sufficient lactate transport is significantly reliant on this synergistic effect. Aberrant CD147 expression will affect the activities of MCTs, modifying intracellular lactate content and transport efficiency, hence influencing the extent of lactylation (52, 53).

#### 3.2.2 Regulation of lactylation by “writer enzymes” and “eraser enzymes”

Histone acetyltransferases (HATs), which catalyze the transfer of lactyl-CoA, are the primary “writer enzymes” involved in the enzymatic process of lactylation. Since they are extremely similar proteins, CBP (CREB-binding protein) and p300 (E1A-associated 300 kDa protein) are frequently referred to as CBP/p300. For numerous transcription factors, CBP/p300 acts as a co-activator. According to research, CBP/p300 can function as a “writer enzyme” during the enzymatic process of lactylation of proteins (48). Under specific circumstances, they can transfer the lactyl group on lactyl-CoA to the lysine residues of proteins utilizing their catalytic domains, resulting in lactylation modification of proteins (54). The degree of lactylation can be significantly impacted by changes in the expression level of CBP/p300 (55). Kla rises in human embryonic kidney 293T cells (HEK293T) when p300 is overexpressed. This suggests that the lactylation reaction can be facilitated and the amount of lactylation raised by up-regulating the expression of CBP/p300 (56, 57). On the other hand, histone lactylation is reduced in human colon carcinoma 116 (HCT116) cells and HEK293T cells when p300 is knocked down (58).

Histone deacetylases 1-3 (HDAC1-3) are referred to as “eraser enzymes” (59) because they can remove lactate groups from proteins and have delactylating action. It is possible to alter the lactylation status of proteins by controlling the actions of these enzymes. They alter the chromatin state and control gene expression by removing the lactylation modification on histones during the regulation of gene expression in cells (60). For instance, HDAC1–3 may be activated to eliminate the lactylation modification on histones, compacting the chromatin structure and inhibiting gene expression when cells need to lower gene transcription activity.

Notably, CBP/p300 and HDAC1–3 exhibit opposing roles in tumor immunity (61) (62). In ovarian cancer, CBP/p300 overexpression correlates with enhanced H3K18 lactylation and aggressive glycolysis, while HDAC1 downregulation in colorectal cancer promotes Treg cell-mediated immunosuppression via sustained histone lactylation (63). This reciprocal regulation highlights their potential as therapeutic targets for rebalancing tumor metabolism and immunity. Beyond ovarian and colorectal cancers (64), CBP/p300 and HDAC1–3 exhibit context-dependent roles in multiple malignancies. In breast cancer (65), CBP/p300-driven lactylation of FOXP3 enhances Treg suppressive function via TGF-β/Smad3 signaling, while HDAC1 downregulation correlates with M2 macrophage infiltration (66). In hepatocellular carcinoma, CBP/p300 cooperates with HIF-1αto lactylate HK2 and VEGF, promoting glycolysis and angiogenesis, whereas HDAC1 activation restores lactate efflux balance to sensitize tumors to immunotherapy.

Notably, in NSCLC (67), HDAC3 loss sustains H3K18 lactylation at the PD-L1 promoter, driving immune checkpoint upregulation (68). These findings highlight the dual roles of lactylation regulators as both metabolic sensors and epigenetic drivers, with implications for precision oncology. For example, the CBP/p300 inhibitor CPI-0610 shows promise in preclinical models of pancreatic cancer by disrupting the lactylation-TTK/BUB1B feedback loop, while HDAC1 agonist entinostat reverses immunosuppression in colorectal cancer by de-repressing CD8+ T cell infiltration.

Controversies persist regarding lactylation specificity. Although most lactylation events rely on lactate as a substrate, recent studies identify lactate-independent pathways, such as the generation of lactyl-CoA from non-glycolytic sources like amino acid metabolism. Additionally, lysine residues modified by lactylation (e.g., H3K18) can compete with acetylation for stoichiometric occupancy. In macrophages, acidic TME conditions favor lactylation over acetylation at shared sites, potentially reprogramming gene expression toward immunosuppression. These findings highlight lactylation as a context-sensitive modification with nuanced regulatory mechanisms.

#### 3.2.3 Activation of signaling pathways promotes lactylation modification

Activation of signaling pathway is the core driving force of lactate modification by regulating lactate metabolism and modifying enzyme activity. It has been found that the regulation of lactate level involves several pivotal signaling pathways, such as HIF-1α pathway, mTOR pathway and PI3K/Akt signaling pathway. The hypoxia-inducing factor HIF-1α is activated under hypoxia, and the expression of glycolytic related genes (LDHA, GLUT1, etc.) is up-regulated, which promotes the production of lactic acid. Previous studies have shown that HIF-1α can also influence intracellular lactate levels by regulating the activity of lactate transporters (such as MCT1), indirectly driving histone lactate modification. When the mTOR (69)pathway is activated, the body promotes glycolysis and inhibits oxidative phosphorylation, which aggravates lactic acid accumulation. Some scholars have found that mTORC1 may directly participate in the process of lactation by regulating the activity of lactate modification enzymes (such as lactate transferase). Glycolysis, lactate generation, and lactylation modification can all be enhanced by activation of the PI3K/Akt pathway, which can also boost the expression and translocation of glucose transporters and the cell’s absorption of glucose (70). The distribution of lactate and the lactylation process can also be further controlled by the PI3K/Akt pathway, which can further control the expression and function of lactate transporters.

Recent advances reveal intricate crosstalk between HIF-1α, mTOR, and PI3K/Akt pathways in regulating lactylation, which is profoundly shaped by tumor microenvironmental contexts. HIF-1α acts as a central hub, not only enhancing glycolysis and lactate production but also upregulating MCT1 to boost intracellular lactyl-CoA availability for lactylation (71). Under hypoxia, HIF-1α suppresses mTORC1 to redirect metabolism toward glycolysis, while in normoxic regions, mTORC1 phosphorylates CBP/p300 at Ser1834 to enhance its lactyltransferase activity, preferentially modifying non-histone proteins like FOXP3 in Tregs (72).

The PI3K/Akt pathway reinforces this network by phosphorylating HK2 and HIF-1α, simultaneously promoting glycolysis and lactylation while inhibiting HDAC3-mediated delactylation. For example, in colorectal cancer, PI3K/Akt-driven β-catenin lactylation at K49 enhances its interaction with TCF/LEF transcription factors, upregulating MCT4 and PD-L1 to form a pro-tumorigenic feedback loop (73). These findings highlight a ‘context-switching’ mechanism where HIF-1α and mTOR/PI3K/Akt pathways dominate in hypoxic vs. normoxic niches, respectively, to fine-tune lactylation for tumor adaptation and immune evasion.

## 4 Glucose metabolic reprogramming and lactylation

Tumor cells have markedly modified their energy metabolism to accommodate the requirements of accelerated growth. Genes associated with glycolysis are typically overexpressed in more than 70% of human tumor cells. Lactate significantly influences tumor growth and carcinogenesis, as demonstrated by prior research. A key hallmark of cancer is glycolytic reprogramming, which entails alterations to downstream metabolites, upstream regulatory molecules, and active enzymes. Lactylation influences metabolic reprogramming in cancer since it constitutes a post-translational alteration of proteins utilizing metabolite residues as substrates. Lactylation can directly enhance the expression of genes associated with metabolism, principally acting as a connection between metabolism and epigenetics.

LDHA is an essential enzyme in the process of lactate production. In a pancreatic cancer study, Liang (74) found that the Kla modification of nucleolar and spindle-associated protein 1 (NUSAP1) regulates LDHA expression, establishing a positive feedback loop involving NUSAP1 Kla, LDHA, glycolysis, and lactate. This elucidates the potential rationale for pancreatic cancer’s inclination towards glycolytic metabolism. Glycolytic enzymes (HK-1, PKM2) demonstrated down-regulation, while TCA cycle enzymes (SDHA, IDH3G) revealed up-regulation in their mRNA expressions in non-small-cell lung cancer during *in vitro* assays (75). Histone Kla in the promoter regions of HK-1 and IDH3G governs this regulation. This indicates that lactylation can regulate the expression of genes associated with metabolism, potentially leading to glycolytic reprogramming and tumor proliferation. Lactate induces dynamic alterations in chromatin, so altering its structure and function, ultimately affecting the expression of genes associated with metabolism. Moreover, lactylation may occur in enzymes associated with metabolic pathways. In clear cell renal cell carcinoma (ccRCC), PDGFRβ signaling can facilitate histone lactylation, establishing an oncogenic positive feedback loop (76). The substantial role of lactate-mediated post-translational modifications in malignancies is further evidenced by the decrease in malignancy noted when lactylation in ccRCC tumor cells is disrupted. Glycolysis and lactylation are thus interconnected and interdependent.

### 4.1 Glucose metabolic reprogramming induces lactylation

Glucose metabolic reprogramming, through the regulation of hypoxia-inducible transcription factor (HIF-1α), STAT, c-Myc, ERK, and other factors, affects lactate production and subsequently the lactylation modification process. HIF-1α facilitates lactate production by modulating the glycolytic pathway via its interaction with the hypoxia-response element (HRE) (77). HRE is located in the promoter regions of various genes associated with glycolysis in tumor cells. HIF-1α possesses binding sites on the promoters of several genes, such as LDHA, PKM2, HK2, and GLUT1 (78). HIF-1α will translocate to the nucleus and bind to the hypoxia response elements (HRE) in the promoter regions of these genes through its DNA-binding domain when cells experience hypoxia or other conditions that activate HIF-1α (79). Moreover, signal transducer and activator of transcription 3 (STAT3) is capable of interacting with HIF-1α. Furthermore, STAT3 is essential for regulating glycolysis in neoplastic cells. It can regulate the expression of genes associated with glycolysis, including PKM2 (80).

After their intricate assembly, HIF-1α and STAT3 collaborate to attach to the promoter regions of genes such as LDHA, thereby enhancing their transcriptional activity. This enhances glycolysis by elevating LDHA expression and promoting the conversion of pyruvate to lactate (81). Lysine lactylation of nucleolar and spindle-associated protein 1 (NUSAP1) by CBP/p300 in pancreatic ductal adenocarcinoma (PDAC) enhances its association with the LDHA promoter, hence increasing LDHA expression. This creates a feedforward loop in which elevated LDHA promotes glycolysis and lactate synthesis, hence supplying additional substrate for NUSAP1 lactylation. Simultaneously, lactate-induced acidity of the TME promotes M2 polarization of tumor-associated macrophages (TAMs), hence enhancing tumor invasion and immune evasion.

Furthermore, HIF-1α and c-Myc engage in a cooperative interaction that regulates one another (82). HIF-1α and c-Myc collaborate to regulate the transcription of genes associated with glycolysis in neoplastic cells. In clear cell renal cell carcinoma (ccRCC), the inactivation of the von Hippel-Lindau (VHL) tumor suppressor stimulates PDGFRβsignaling, subsequently promoting histone lactylation through CBP/p300. Lactylated histones at oncogenic loci (e.g., c-Myc, HIF-1α) initiate a positive feedback loop with glycolytic enzymes, augmenting lactate generation and MCT4-mediated lactate export. The interruption of this loop with lactylation inhibitors diminishes ccRCC cell proliferation and tumor formation in preclinical models, underscoring its therapeutic promise. They can, for example, collaboratively bind to the promoter of the HK2 gene, thereby enhancing HK2 transcription (83). An elevation in the activity of HK2, an essential enzyme in glycolysis, may promote the phosphorylation of glucose and expedite the glycolytic process (74). In NSCLC, histone lactylation (e.g., H3K18la) in the promoter regions of glycolytic genes (HK-1) and TCA cycle genes (IDH3G) modifies their transcriptional activity. The downregulation of HK-1 through diminished lactylation restricts glycolysis, but the overexpression of IDH3G enhances TCA cycle flux, illustrating context-dependent metabolic reprogramming. This epigenetic interaction is essential for NSCLC cell tolerance to hypoxia and resistance to metabolic-targeted treatments. The proliferation and incidence of malignancies are greatly affected by members of the MAPK family, particularly extracellular signal-regulated kinase (ERK). HIF-1α is subject to phosphorylation by ERK, enhancing its transcriptional activity and facilitating nuclear translocation. The MAPK pathway might indirectly affect the transcriptional regulation of HIF-1α on glycolysis-related genes by modulating other transcription factors or signaling molecules. PKM2, an essential enzyme in glycolysis and a transcriptional co-activator, can have its expression upregulated by the MAPK pathway, which subsequently promotes the transcription of glycolysis-associated genes (84). Consequently, HIF-1α is crucial to the lactylation process. In conclusion, the promotion of cellular conversion to glycolysis can regulate lactylation through hypoxia, inactivation of tumor suppressor factors, and activation of oncogenes.

### 4.2 Regulation of glucose metabolic reprogramming by lactylation modification

Lactylation modification primarily influences glucose metabolic reprogramming by affecting the structural functions and transcriptional expression of key glycolytic proteins. Lactate suppresses tumor cell glycolysis by facilitating the ubiquitination and degradation of PFKFB3, an essential regulator of glycolysis, through the APC/C-Cdh1 pathway (85). Lactate acts as a substrate to promote the lactylation modification of histone H3K18 in pancreatic ductal carcinoma. The transcription of TTK protein kinase and BUB1B can be augmented by H3K18la, whereas P300 can be upregulated by TTK and BUB1B. TTK can activate lactate dehydrogenase A to enhance lactate generation and facilitate histone lactylation. This establishes a positive feedback loop of glycolysis involving H3K18la and TTK/BUB1B, perpetually stimulating glycolysis and tumor proliferation (76). Lactate may influence the glucose metabolism of macrophages.

Previous study indicates that tumor cells can transform tumor-associated macrophages (TAMs) toward an M2 immunosuppressive phenotype. Lactate, a vital signaling molecule, modulates the metabolic reprogramming of macrophages, inhibiting immune responses and promoting tumor growth (85). TME is saturated with lactate produced during glycolysis, which can influence macrophages attracted to TME, particularly in their detection and binding by GPR132 (86). Lactate can stabilize HIF-1α and enhance the expression of M2 genes, such as PPAR-γ, arginase-1, Fizz-1, Mgl-1, and VEGF. It promotes tumor development, invasion, and metastasis by polarizing tumor-associated macrophages into an M2 immunosuppressive phenotype (32). The chemical mechanism by which lactate modulates glycolysis in macrophages has recently been elucidated by lactylation modification.

### 4.3 Tumor immune escape mediated by lactylation and glucose metabolic reprogramming

Glucose metabolism reprogramming in the TME systematically reshapes immune cell functions through lactate production and lactylation modification, establishing a multidimensional immune evasion network.

This reprogramming synergistically suppresses the antitumor activity of T cells by both nutrient deprivation and lactylation-mediated regulation. Tumor cells exhibit high expression of GLUT1, which reduces extracellular glucose levels to ≤0.5 mM in the microenvironment (87). This leads to a 50% decrease in glycolytic rate in CD8+ T cells, insufficient mitochondrial ATP production, and subsequent failure to maintain proliferation and cytotoxic granule secretion (88). Concurrently, regulatory T cells (Tregs) prioritize survival in low-glucose conditions through high GLUT3 expression and activated mitochondrial oxidative metabolism. Lactylation modification at the K277 site of FOXP3 protein enhances its DNA-binding capacity, significantly upregulating the secretion of IL-10 and TGF-β to inhibit effector T cell function (89). Lactylation further sustains T cell exhaustion via epigenetic mechanisms: lactate (≥5 mM) inhibits HDAC3 activity, increasing H3K18 lactylation at the PD-1 promoter by 2.1-fold in CD8+ T cells and perpetuating PD-1 expression (90).

Macrophage phenotype switching is coordinately regulated by glucose metabolism reprogramming and lactylation. Pro-inflammatory M1 macrophages rely on robust glycolysis (with GLUT1/HK2 activity threefold higher than that of M2 macrophages), generating abundant lactate and succinate to drive HIF-1α-mediated pro-inflammatory gene expression. However, lactate accumulation (>10 mM) in the TME forces macrophages to shift toward oxidative phosphorylation, activating the PPAR-γ/CD36 pathway for fatty acid uptake. Simultaneously, CBP/p300-mediated H3K18 lactylation enriches at the Arg1 promoter, stabilizing the immunosuppressive M2 phenotype. Lactylation reinforces pro-tumor macrophage functions through positive feedback loops: lactylation at the K316 site of glycolytic enzyme LDHA enhances its activity by 1.8-fold, further promoting lactate production (91); lactylation at the K116 site of TREM2 in tumor-associated macrophages (TAMs) strengthens its interaction with tumor cell CD44, facilitating the deposition of immunosuppressive extracellular matrix and inducing immune tolerance.

Dendritic cell (DC) maturation and antigen presentation depend on energy provided by glucose metabolism (92). However, glucose deprivation (<1 mM) in the TME prevents DCs from upregulating GLUT1, leading to UDPG deficiency and glycosylation defects in MHC class II molecules, which become trapped in the endoplasmic reticulum. Lactate further disrupts the tricarboxylic acid (TCA) cycle by inhibiting mitochondrial pyruvate carriers (MPCs), causing α-ketoglutarate depletion and suppressing histone demethylase activity. This results in enrichment of H3K27me3 modifications at the promoters of antigen presentation-related genes (e.g., CIITA). For example, reduced HDAC2 expression in DCs from colorectal cancer leads to aberrant accumulation of H3K18 lactylation at CD80/CD86 promoters, suppressing costimulatory molecule expression and reducing CD4+ T cell activation efficiency by 90%. Metabolites from the pentose phosphate pathway inhibit their binding to the IFN-β promoter via lactylation, blocking innate immune responses in DCs.

The interplay between glucose metabolism reprogramming and lactylation offers multifaceted targets for tumor immunotherapy. For instance, inhibiting HDAC3 reduces lactylation at the PD-1 promoter and restores CD8+ T cell function; targeting CBP/p300 blocks M2 polarization of macrophages and promotes pro-inflammatory phenotype switching. Lactylation inhibitors for natural killer (NK) cells and HDAC2 activators for DCs have also demonstrated potential in preclinical models to restore immune surveillance.

## 5 Conclusion and prospects

The homeostasis of rapidly growing tumor cells is closely associated with glycolytic reprogramming. Alongside the swift provision of energy, alterations in glucose metabolism are essential for maintaining intracellular redox balance. Lactate is a metabolic byproduct of glucose, and its accumulation promotes lactylation, a distinct post-translational modification of proteins. Lactylation strongly influences various physiological and pathological processes, including tumor formation, inflammation, and immune regulation. Despite hypoxia, OXPHOS reduction, or elevated glucose consumption significantly enhancing histone lactylation, further investigation is required to elucidate the mechanism connecting lactylation with glycolysis. Lactylation, frequently linked to lactate buildup, is becoming recognized as a causal epigenetic modulator. For example, the overexpression of CBP/p300 in HEK293T cells promotes histone lactylation even in low lactate environments, demonstrating enzyme-driven selectivity. Inhibition of HDAC1–3 increases lactylation levels regardless of lactate availability, indicating that the equilibrium between ‘writer’ and ‘eraser’ enzymes, rather than lactate alone, governs lactylation dynamics. This dual nature underscores the intricacy of lactation. Our investigation of lactylation as an innovative post-translational alteration remains in its preliminary phases. Although new study suggests a correlation between lactate and lactylation, it remains uncertain if lactylation consistently occurs with elevated lactate levels. The lack of specialized “writer” and “eraser” enzymes complicates the regulation of histone lactylation. Consequently, further research is required to develop specific inhibitors for histone lactylation and to further comprehension of the mechanisms governing lactylation. Lactate directly elevates immunosuppressive molecules such as PD-L1 in tumor cells by altering histone or non-histone proteins, while acidification of the microenvironment inhibits the functionality of T and NK cells. Targeted lactation can enhance the anti-tumor response and alter the immune microenvironment (93).

Inhibiting the monocarboxylic acid transporter MCT1/4 can obstruct lactic acid efflux and reduce lactate levels in TME (93, 94); targeting the pivotal glycolytic enzymes LDHA or HK2 can diminish lactic acid synthesis; and integrating these approaches with PD-1/PD-L1 inhibitors, CAR-T (95), and similar therapies can restore T cell functionality and enhance therapeutic efficacy. Focusing on lactylation-related pathways presents potential for cancer treatment. MCT1 inhibitors (e.g., AZD3965) are currently undergoing Phase II trials for pancreatic and breast cancer, with the objective of obstructing lactate export and acidifying TME to rejuvenate T cell functionality. New LDHA allosteric inhibitors (e.g., FX-11) demonstrate preclinical effectiveness in diminishing glycolytic flux and lactylation levels, especially in malignancies reliant on the Warburg effect. The integration of these drugs with immune checkpoint inhibitors (e.g., anti-PD-1) or CAR-T cell therapy may synergistically counteract immunosuppression by interrupting lactate-induced M2 macrophage polarization and augmenting T cell infiltration. These developments connect fundamental research on metabolic-epigenetic interactions with clinical applications, highlighting the translational potential of lactylation-focused treatments. Lactylation provides a novel perspective and holds significant potential for tumor diagnostics and therapeutic strategies as a connection between metabolism and epigenetics.
